# Analysis of differences between ^99m^Tc-MAA SPECT- and ^90^Y-microsphere PET-based dosimetry for hepatocellular carcinoma selective internal radiation therapy

**DOI:** 10.1186/s13550-019-0533-6

**Published:** 2019-07-22

**Authors:** Marilyne Kafrouni, Carole Allimant, Marjolaine Fourcade, Sébastien Vauclin, Boris Guiu, Denis Mariano-Goulart, Fayçal Ben Bouallègue

**Affiliations:** 1 Department of Nuclear Medicine, Montpellier University Hospital, Montpellier, France; 2 PhyMedExp, Montpellier University, INSERM, CNRS, Montpellier, France; 3 DOSIsoft SA, Cachan, France; 4 Department of Radiology, Montpellier University Hospital, Montpellier, France

**Keywords:** MAA, Y-90 microspheres, SIRT, Dosimetry, Hepatocellular carcinoma

## Abstract

**Background:**

The aim of this study was to compare predictive and post-treatment dosimetry and analyze the differences, investigating factors related to activity preparation and delivery, imaging modality used, and interventional radiology.

**Methods:**

Twenty-three HCC patients treated by selective internal radiation therapy with ^90^Y glass microspheres were included in this study. Predictive and post-treatment dosimetry were calculated at the voxel level based on ^99m^Tc-MAA SPECT/CT and ^90^Y-microsphere PET/CT respectively. Dose distribution was analyzed through mean dose, metrics extracted from dose-volume histograms, and Dice similarity coefficients applied on isodoses. Reproducibility of the radiological gesture and its influence on dose deviation was evaluated.

**Results:**

^90^Y delivered activity was lower than expected in 67% (16/24) of the cases mainly due to the residual activity. A mean deviation of − 6 ± 11% was observed between the delivered activity and the ^90^Y PET’s FOV activity. In addition, a substantial difference of − 20 ± 8% was measured on ^90^Y PET images between the activity in the liver and in the whole FOV. After normalization, ^99m^Tc-MAA SPECT dosimetry was highly correlated and concordant with ^90^Y-microsphere PET dosimetry for all dose metrics evaluated (*ρ* = 0.87, *ρ*_c_ = 0.86, *P* = 3.10^−8^ and *ρ* = 0.91, *ρ*_c_ = 0.90, *P* = 7.10^−10^ for tumor and normal liver mean dose respectively for example). Besides, mean tumor dose deviation was lower when the catheter position was identical than when it differed (16 Gy vs. 37 Gy, *P* = 0.007). Concordance between predictive and post-treatment dosimetry, evaluated with Dice similarity coefficients applied on isodoses, significantly correlated with the distance of the catheter position from artery bifurcation (*P* = 0.04, 0.0004, and 0.05, for 50 Gy, 100 Gy, and 150 Gy isodoses respectively).

**Conclusions:**

Discrepancies between planned activity and activity measured on ^90^Y PET images were observed and seemed to be mainly related to clinical hazards and equipment issues. Predictive vs. post-treatment comparison of relative dose distributions between tumor and normal liver showed a good correlation and no significant difference highlighting the predictive value of ^99m^Tc MAA SPECT/CT-based dosimetry. Besides, the reproducibility of catheter tip position appears critical in the agreement between predictive and actual dose distribution.

## Background

Selective internal radiation therapy (SIRT) is a valuable locoregional therapeutic option for inoperable hepatocellular carcinoma (HCC). Injection of ^99m^Tc-labeled macroaggregated albumin (MAA) followed by planar scintigraphy and SPECT acquisitions prior to therapy (referred to as the “simulation” phase) is part of the SIRT general procedure. The aim is to assess lung shunt fraction, detect any extrahepatic uptake, and predict ^90^Y-microsphere distribution. However, although largely discussed in the literature [1–6], the ability of the ^99m^Tc-MAA simulation to predict actual ^90^Y-microsphere therapy is still debated.

Interest for activity planning using MAA-based personalized dosimetry is growing in SIRT [2, 6, 7]. Physical property differences between MAA and microspheres (size/shape, density, amount of particles injected, etc.) [1], the two-stage procedure, and the different imaging modalities used lead to expect variations in dosimetry estimations. A few studies have addressed this issue so far. Gnesin et al. compared predictive and delivered doses to the tumor and normal liver (NL) calculated at the voxel level based on the ^99m^Tc-MAA SPECT/CT and ^90^Y-microsphere PET/CT for both glass and resin microsphere SIRT [5]. They concluded that the predictive dose based on the ^99m^Tc-MAA SPECT/CT is a valuable predictor of post-treatment dosimetry with discrepancies in some specific patient cases. Contrariwise, Haste et al. concluded on a patient cohort treated with glass microspheres that ^99m^Tc-MAA SPECT/CT is a poor predictor of ^90^Y-microsphere tumor dose but can be used for NL dose prediction [3]. Song et al. used the partition model on a population treated with resin microsphere, to report a good correlation between pre- and post-treatment doses despite significant differences, and suggested to use MAA planning as a conservative planning method [4]. According to Gnesin et al. and Song et al., discrepancies between pre- and post-treatment dose estimates may be attributed to different factors which respective influence remain unclear: flow differences between MAA and microspheres, catheter position deviations, differences between imaging modalities used, etc.

The aim of this study was to analyze the differences between ^99m^Tc-MAA SPECT/CT and ^90^Y-microsphere PET/CT dosimetry, investigating factors related to activity preparation and delivery, imaging modality used, and interventional radiology, based on an HCC patient cohort treated with glass microspheres.

## Methods

### Patient characteristics

Twenty-three unresectable HCC patients treated at our institution by SIRT using ^90^Y glass microspheres from October 2015 to September 2018 were considered for this retrospective study. Among them, nine and four patients were included in DOSISPHERE and STOPHCC trials respectively [8, 9]. Authorization for the ancillary study was obtained from the principal investigators.

All the patients received the injection of microspheres in a single session except for one patient who underwent two sequential SIRT due to a reflux during injection at the first session. For this ^99m^Tc-MAA/^90^Y-microsphere dosimetry comparison, these two sessions were considered as distinct procedures. All selected patients were at an intermediate or advanced stage of the disease. Baseline characteristics of the patients included are summarized in Table 1.

**Table 1:** Baseline characteristics for the 23 patients

Clinical variable	Value
Age (years)	63 ± 9
Sex (*n*)	
Male	21
Female	2
Child-Pugh class* (*n*)	
A5	19
A6	2
B7	1
BCLC stage (*n*)	
B	2
C	21
Prior local therapy** (*n*)	
Yes	6
No	17
Tumor morphology (*n*)	
Infiltrative	15
Nodular	8
Portal vein thrombosis (*n*)	
Yes	20
No	3
Total tumor volume (mL)***	
Mean ± SD	437 ± 344
Median [range]	353 [58–1250]
Liver tumor involvement (*n*)	
< 25%	15
25–50%	8
Treatment (*n* + 1)	
Whole liver	1
Lobar	17
Sectorial	4
Segmental	2

*WHO* World Health Organization, *BCLC* Barcelona Clinic Liver Cancer

*One patient was non-cirrhotic

**Prior local therapies include chemoembolization and radiofrequency ablation

***Number of lesions was 1 for all treatments except one treatment that concerned 2 lesions

### General procedure

SIRT was applied following the general procedure described in the literature [10]. Prior to the treatment, an angiography was performed for hepatic vasculature mapping, potential coil embolization of extrahepatic vessels, and determination of optimal catheter position. This was followed by injection of a standardized activity of 185 MBq of ^99m^Tc-MAA and acquisition within an hour of planar images for assessment of lung shunting and SPECT/CT for visualization of potential extrahepatic microsphere deposition and tumor targeting and for activity planning. For patients included in trials, the activity to be delivered was planned according to the protocol instructions, i.e., for DOSISPHERE trial, delivering 120 ± 20 Gy to the treated liver or more than 205 Gy to the tumor (standard vs. optimized dosimetry arm [8]), and for STOPHCC trial, delivering 120 Gy ± 10% to the treated lobe of the liver [9]. For patients not included in any trials, activity was planned using personalized dosimetry with a 205 Gy minimum mean dose objective to the tumor [2] and a maximum mean dose of 50 Gy and 30 Gy to the NL and to the lungs respectively. Doses were assessed on the ^99m^Tc-MAA SPECT images using a voxel-based approach detailed in the dosimetry paragraph.^90^Y glass microspheres (TheraSphere®, BTG Biocompatibles Ltd., Farnham, UK) were ordered through the form provided by the manufacturer including an estimated 2% residual activity as a preventive measure. ^90^Y-microspheres were administered 18 ± 7 days after the simulation stage (range 12–37 days) according to the manufacturer’s instructions. Before injection, the ^90^Y activity in the microsphere vial was systematically measured with the dose calibrator. Residual activity in the vial and the radiology material used was systematically assessed following the manufacturer recommendations [11], i.e., considering the ratio of the mean dose rate measured at four 90°-spaced points after and before injection. This ratio was then applied to the vial activity measured before injection to deduce the residual activity. The delivered activity, defined as the subtraction between the vial activity before injection and the residual activity, was 3.6 ± 1.2 GBq with a range of 0.9–6.6 GBq. ^90^Y PET/CT images were acquired on the following day.

### Imaging

SPECT/CT data were acquired on a Symbia Intevo system (Siemens Healthcare, Erlangen, Germany) with the following parameters: window of 140 keV ± 7.5%, 32 projections per head, 25 s/projection, matrix 128 × 128, voxel size 4.79 mm × 4.79 mm × 4.79 mm, and low energy collimator. SPECT/CT data were reconstructed with Flash 3D Iterative Reconstruction was applied using 5 iterations/8 subsets, 6 mm Gaussian filter, with attenuation correction using a CT attenuation map, and scatter correction applying the Jaszczak method (dual-energy-window scatter correction, with a scatter window of 120 keV ± 7.5%, weighting factor of 0.5).

PET/CT data were obtained on a Biograph mCT flow (Siemens) with liver-centered continuous bed motion image acquisitions (bed speed of 0.2 mm/s). The PET/CT reconstruction parameters used for SIRT dosimetry were TrueX + time of flight reconstruction algorithm (Siemens), all-pass filter, 2 iterations, 21 subsets, and matrix 200 × 200 with voxel size 4.07 × 4.07 × 2.03 mm^3^. ^90^Y PET data were corrected for attenuation and scatter using the single scatter simulation method. The PET scanner was calibrated by the manufacturer to measure ^90^Y emission quantitatively. Moreover, this was verified beforehand applying the QUEST study by Willowson et al. using a NEMA 2007/IEC 2008 PET Body Phantom (Data Spectrum Corporation, NC) [12].

### Dosimetry

Predictive and post-treatment dose calculations were carried out based on the ^99m^Tc-MAA SPECT/CT and ^90^Y-microsphere PET/CT images respectively using a dedicated software (PLANET® Dose, DOSIsoft SA, Cachan, France). The general workflow applied was similar to the one described in a previous study [13]. Briefly, tumor and NL were defined manually by an expert radiologist using prior morphologic imaging data (contrast-enhanced CT or magnetic resonance imaging). Only lesions larger than 2 cm located in the targeted lobe were considered in order to limit bias induced by partial-volume effect. The number of lesions was 1 for all treatments except for one treatment that concerned 2 lesions. ^99m^Tc-MAA SPECT/CT and ^90^Y-microsphere PET/CT were rigidly co-registered with the imaging exam used for volume delineation. Thus, the same tumor and NL contours were used for both ^99m^Tc-MAA SPECT/CT- and ^90^Y-microsphere PET/CT-based dose calculations. Three-dimensional dose maps at the voxel level were calculated for predictive and post-treatment dosimetry using a Voxel S-Values dose kernel convolution algorithm. Post-treatment dosimetry was performed using the activity concentration directly quantified on ^90^Y PET data; no other calibration factor was applied.

### Planned vs. delivered vs. measured activity

On the one hand, the ^90^Y planned activity was compared to the delivered activity to include all the clinical hazards: vial selection, actual time of injection vs. expected time, and residual activity. On the other hand, the activities measured in the whole field of view (FOV) and in the anatomically segmented liver on PET images were compared to the planned and delivered activity.

### Dose distribution relative difference

In order to assess dosimetric discrepancies related to differences in dose spatial distribution, ^99m^Tc-MAA SPECT was normalized so that the liver activity corresponds to that quantified inside the liver on ^90^Y PET (designated as normalized ^99m^Tc-MAA SPECT).

For each treatment, data related to radiological gesture were compared by a single expert radiologist between simulation and treatment stages using patient records and angiographic images: operator, radiology material used, catheter position, distance to major bifurcation, volumes of injection, and potential vascularization modifications. The difference of catheter tip position was considered when a deviation > 5 mm was measured between simulation and therapy angiographic data. Distance to major bifurcation was estimated on dynamic planar (11/24) or CT (13/24) angiographic data when available and classified as follows: ≤ 5 mm, 10 ± 2 mm, 15 ± 2 mm, 20 ± 2 mm, > 22 mm considering the uncertainty of measurement.

The following metrics extracted from ^90^Y-microsphere PET and normalized ^99m^Tc-MAA SPECT dosimetry were used for comparison: mean dose to the tumor (*D*_T_) and to the NL (*D*_NL_), as well as dose-volume histogram-based minimal dose to 70%, 50%, and 20% of the tumor volume (*D*_70_, *D*_50_, and *D*_20_ respectively) and percentage of the tumor volume receiving at least 205 Gy (*V*_205_).

Isodose volumes from normalized predictive and post-treatment dosimetry were compared using the Dice similarity coefficient [14]. Assessed isodoses corresponded to 50, 100, and 150 Gy referred as DC_50_, DC_100_, and DC_150_ respectively.

### Statistical analysis

Dose metrics based on ^99m^Tc-MAA SPECT and ^90^Y-microsphere PET were compared using paired Student’s *t* tests. Pearson’s correlation coefficient (*ρ*), Bland-Altman analysis, and Lin’s concordance coefficient (*ρ*_c_) were used to evaluate the agreement between predictive and post-treatment dosimetry. The normality of the data distributions was checked using the Kolmogorov-Smirnov test. Pearson’s correlation coefficient (*ρ*) was also used to evaluate the correlation between ^90^Y PET activity recovery and patient’s BMI.

Predictive vs. post-treatment dose disparity was measured through the absolute difference for dose metrics (*D*_T_, *D*_NL_, *D*_20_, *D*_50_, *D*_70_, and *V*_205_) and isodose Dice similarity (DC_50_, DC_100_, DC_150_). The following parameters were investigated as potential determinants of predictive vs. post-treatment dose disparity in univariate and multivariate analysis: age, body mass index (BMI), Child-Pugh class, BCLC stage, delay between simulation and treatment, type of tumor (infiltrative vs. nodular), portal vein thrombosis, tumor volume, liver volume, type of targeting (segmental, sectorial, lobar, or whole liver), lung shunt fraction, administered activity, difference between delivered and planned activity, and radiological gesture data (including operator identity, type of material, difference in catheter position, and distance from major bifurcation at treatment). Univariate analysis was performed by testing Pearson’s correlation between the dose disparity metric and the potential explanatory variable. Multivariate analysis was conducted using a forward-stepwise linear regression with an entry criterion of *P* ≤ 0.1 and a removal criterion of *P* > 0.05. Overall, a *P* value of 0.05 or less was considered significant.

## Results

### Planned vs. delivered vs. measured activity

^90^Y delivered activity was lower than expected in 67% (16/24) of the cases. The difference between the planned activity and the one ordered selecting the closest vial was − 69 ± 133 MBq (− 2 ± 4%). Injection time was always later than expected except for two treatments. The delay between the expected and actual time of injection was 89 ± 55 min, resulting in a difference of activity of − 1.6 ± 1.0%. The residual activity measured after ^90^Y-microsphere injection was 6 ± 7% including three cases with a substantial (> 10%) residual activity. The relative deviation between the planned activity and the delivered activity was − 148 ± 491 MBq (− 3 ± 9%).

The planned and delivered therapeutic activities, as well as the activities measured from PET acquisition in the whole FOV (covering mainly the liver) and inside the anatomic liver, are given in Table 2.

**Table 2:** Comparison between the planned therapeutic activity, the delivered therapeutic activity, the activity measured in the PET’s FOV, and the activity in the liver measured from PET

	Mean ± SD	Absolute deviation	Relative deviation
Planned therapeutic activity (MBq)	3673 ± 1387	–	–
Delivered therapeutic activity (MBq)	3525 ± 1225	− 148 ± 491	− 3 ± 9%
Activity measured in the PET’s FOV (MBq)	3301 ± 1162	− 372 ± 534	− 9 ± 12%
Activity in the liver measured from PET (MBq)	2663 ± 1002	− 1010 ± 579	− 27 ± 10%

*SD* standard deviation, *FOV* field of view. All activities are given at the same time of injection. Deviation is computed with respect to the activity planned to be delivered

Difference between the delivered activity and the PET’s FOV activity was − 6 ± 11%. Besides, a substantial difference of − 20 ± 8% could be noticed between the activity measured in the segmented liver on the PET and the total activity in the PET’s FOV. This result was supported by the measurement made using an anthropomorphic phantom [15] (experiments not detailed here) where a deviation of − 15% between the activity in the liver of the phantom and in the total PET’s FOV was observed.

**Fig. 1:**
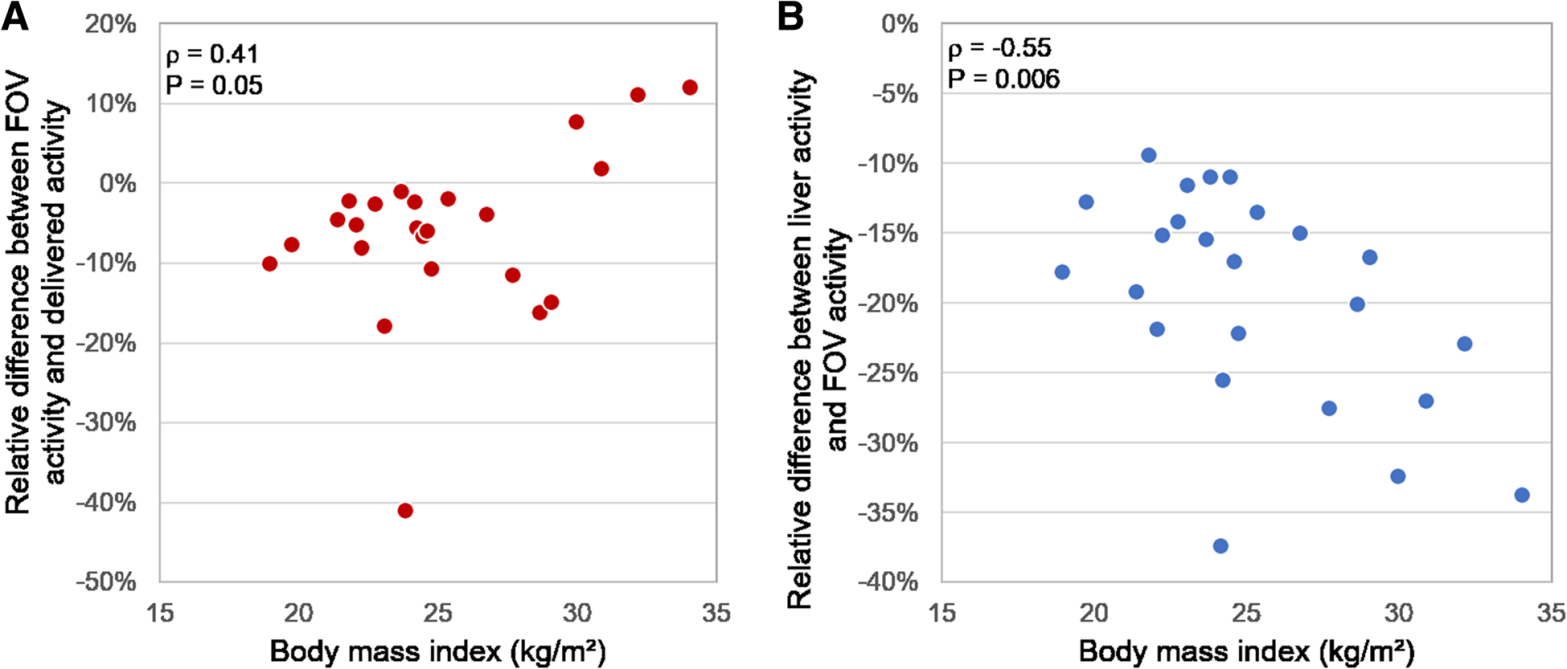
Relative difference between the activity measured in the whole FOV of ^90^Y-PET images and the delivered activity (**a**) and between the activity measured in the segmented liver and the whole FOV of ^90^Y-PET images (**b**), both as a function of BMI

In addition to these results, the two types of differences (activity in the PET’s FOV vs. delivered activity and activity in the segmented liver vs. activity in the PET’s FOV) were correlated to the patient’s BMI (*ρ* = 0.41, *P* = 0.05 and *ρ* = − 0.55, *P* = 0.006 respectively) (Fig. 1).

### Dose distribution relative difference

**Fig. 2:**
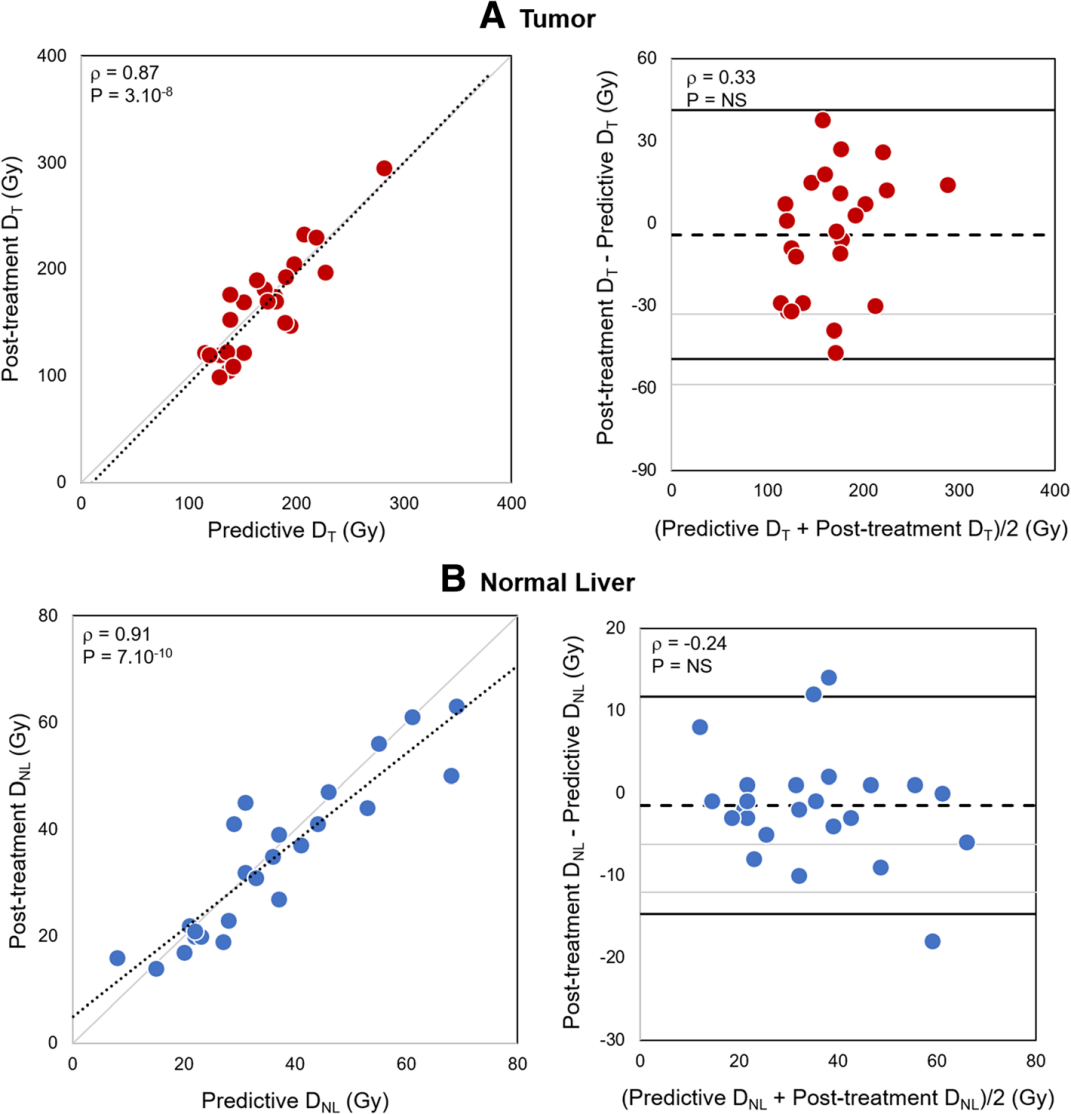
Post-treatment vs. predictive dosimetry based on normalized ^99m^Tc-MAA SPECT and ^90^Y-microsphere PET respectively for tumor (**a**) and normal liver (**b**). Left: scatter plots. The dotted lines stand for the linear regression (*ρ*: Pearson’s correlation). Right: Bland-Altman diagrams. The dashed lines indicate the mean bias (grayed is the 95% confidence interval) and the plain lines the 95% limits of agreement. *D*_T_, mean dose to the tumor; *D*_NL_, mean dose to the normal liver

After normalization, ^99m^Tc-MAA SPECT dosimetry was highly correlated and concordant with ^90^Y-microsphere PET dosimetry for *D*_T_ (*ρ* = 0.87, *ρ*_c_ = 0.86, *P* = 3.10^−8^) and for *D*_NL_ (*ρ* = 0.91, *ρ*_c_ = 0.90, *P* = 7.10^−10^). Bland-Altman plot did not show any correlation between dose deviation and dose value (Fig. 2). No significant difference was found between ^99m^Tc-MAA SPECT and ^90^Y-microsphere PET dose metrics except a minimal difference in *D*_70_ (15 Gy, *P* = 0.04) (Table 3).

**Table 3:** Planned and delivered dose results based on normalized ^99m^Tc-MAA SPECT and ^90^Y-microsphere PET respectively

	Normalized ^99m^Tc-MAA SPECT Mean ± SD	^90^Y-MS PET Mean ± SD	Bias [95% CI]	Pearson’s correlation
*D*_T_ (Gy)	169 ± 40	165 ± 47	4 [−5; 13]	0.87 (*P* = 3.10^−8^)
*D*_20_ (Gy)	250 ± 67	251 ± 78	− 1 [− 15; 14]	0.89 (*P* = 7.10^−9^)
*D*_50_ (Gy)	156 ± 44	144 ± 51	12 [0; 25]	0.79 (*P* = 4.10^−6^)
*D*_70_ (Gy)	104 ± 36	91 ± 38	13 [1; 25]*	0.67 (*P* = 3.10^−4^)
*V*_205_ (%)	30 ± 18	30 ± 16	0 [− 3; 4]	0.85 (*P* = 1.10^−7^)
*D*_NL_ (Gy)	36 ± 16	34 ± 15	1 [− 1; 4]	0.91 (*P* = 6.10^−10^)

*MS* microsphere, *MAA* macroaggregated albumin, *SD* standard deviation, *D*_*T*_ mean dose to the tumor, *D*_*20*_ minimum dose to 20% of the tumor volume, *D*_*50*_ minimum dose to 50% of the tumor volume, *D*_*70*_ minimum dose to 70% of the tumor volume, *V*_*205*_ percentage of the volume receiving more than 205 Gy, *D*_*NL*_ mean dose to the normal liver. Bias and correlation are with respect to ^90^Y-MS PET

*P ≤ 0.05

Details regarding radiological gesture are summarized in Table 4. In four scenarios, a 5-French catheter was used for simulation and a 4-French was used for therapy or vice versa, but the same microcatheter was used for both procedures. In two scenarios, both the catheter and the microcatheter differed between simulation and therapy. Catheter details were missing for one patient. No modification of tumor vascularization was observed between simulation and therapy for the patients included in this study. The injected volume was always different between simulation and therapy: 5 mL of ^99m^Tc-MAA solution vs. 60 mL of ^90^Y-microspheres including rinsing.

**Table 4:** Details of radiological gesture regarding operator, material, and catheter position similarity between simulation and treatment and distance from major bifurcation at treatment

	*N* (%)
Same operator	9/24 (38%)
Same material	16/23* (70%)
Same catheter position	21/24 (88%)
Distance from main artery bifurcation at treatment	
≤ 5 mm	4/24 (17%)
10 ± 2 mm	4/24 (17%)
15 ± 2 mm	3/24 (13%)
20 ± 2 mm	4/24 (17%)
> 22 mm	9/24 (38%)

*Details regarding the material used were missing for one procedure

**Fig. 3:**
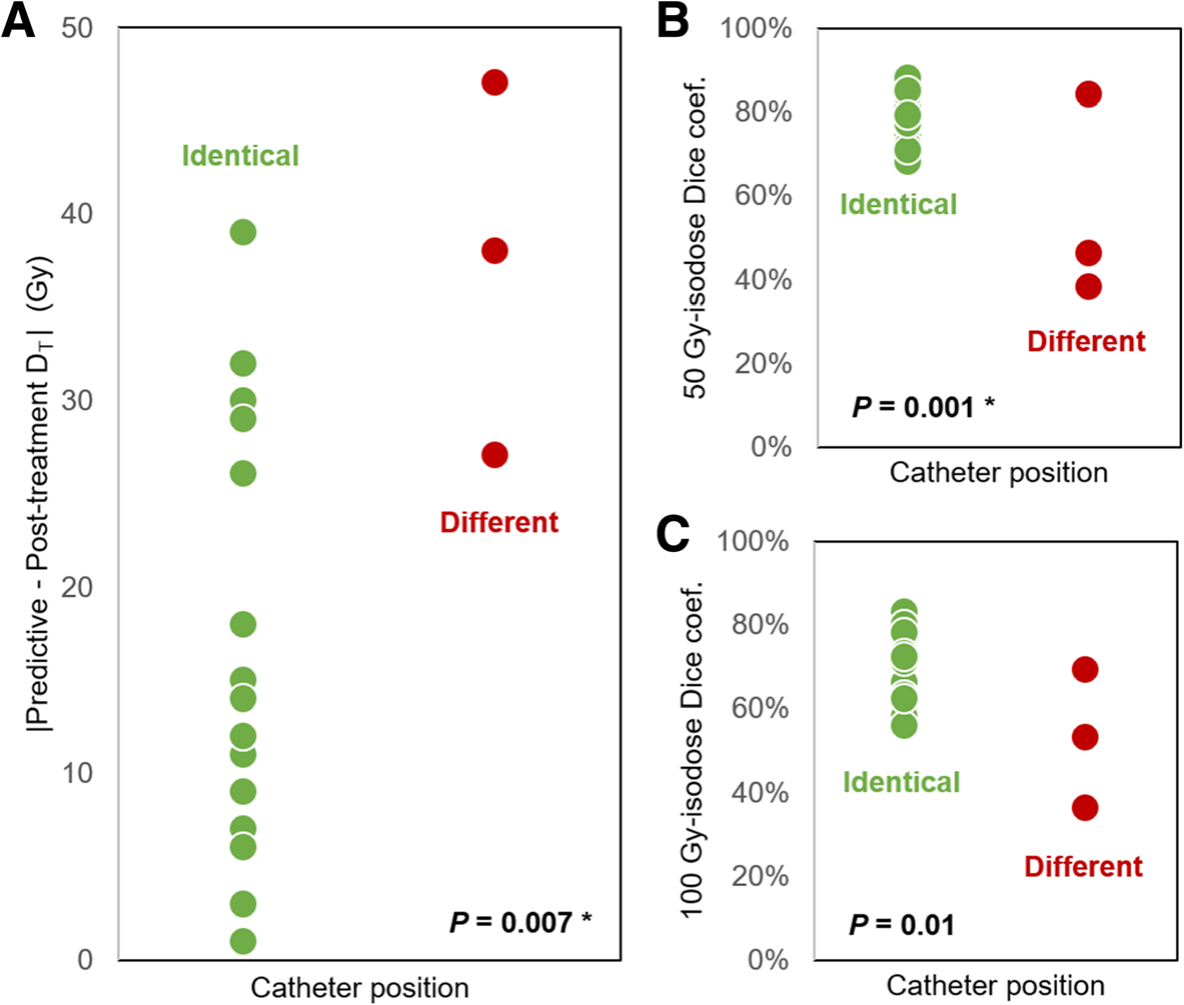
Difference in dose distribution between normalized ^99m^Tc-MAA SPECT and ^90^Y-microsphere PET. **a** Absolute mean dose difference according to the catheter position. Dice coefficient similarity according to the catheter position for 50 Gy isodoses (**b**) and 100 Gy isodoses (**c**). Asterisks (*) indicate *P* values that remained significant in multivariate analysis

Regarding tumor dosimetry, the sole parameter that was found to be significantly associated with *D*_T_ difference in both univariate and multivariate analyses was catheter position between simulation and therapy. Mean dose deviation was lower when the catheter position was identical than when it differed (16 Gy vs. 37 Gy, *P* = 0.007) (Fig. 3a). Besides, *D*_T_ and *D*_50_ difference were negatively correlated with the catheter tip distance from major artery bifurcation (*P* = 0.03 and 0.01 respectively).

**Fig. 4:**
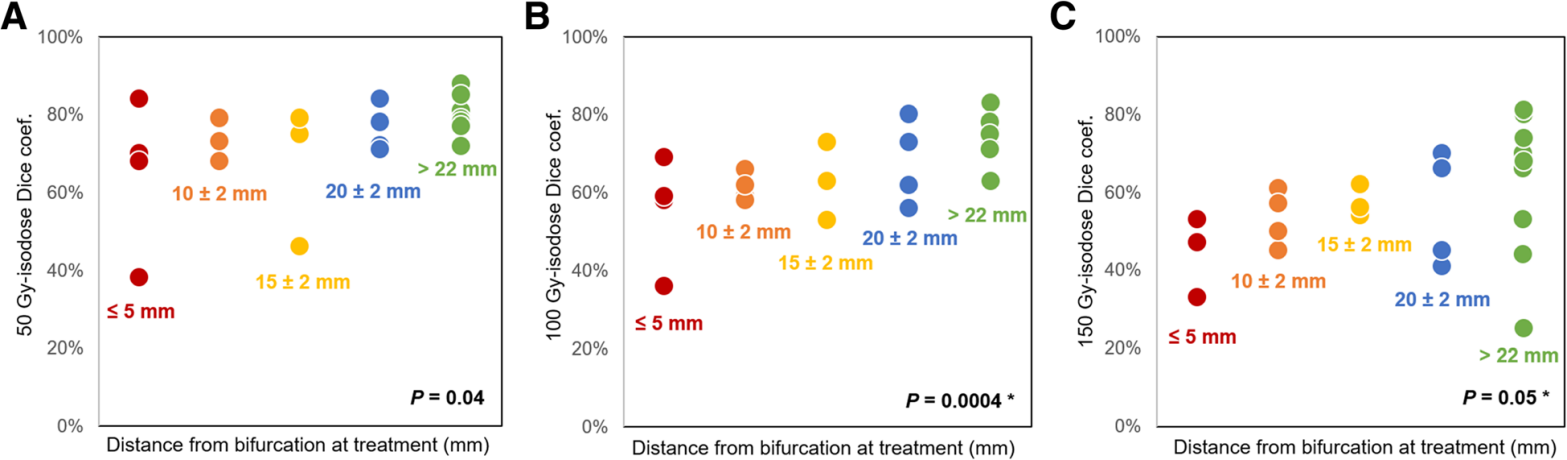
50 Gy (**a**), 100 Gy (**b**), and 150 Gy (**c**) isodose Dice coefficient similarity according to the catheter tip distance from major artery bifurcation at treatment. Asterisk (*) indicates *P* value that remained significant in multivariate analysis

Dice coefficients calculated on the volumes extracted from 50 Gy and 100 Gy isodoses were significantly higher when catheter tip position was identical for simulation and therapy than when it differed (0.77 vs. 0.56; *P* = 0.001, and 0.68 vs. 0.53; *P* = 0.01 for DC_50_ and DC_100_ respectively) (Fig. 3b and c). Spatial concordance between predictive and post-treatment dosimetry also significantly correlated with the distance of the catheter position from artery bifurcation (*P* = 0.04, 0.0004, and 0.05, for DC_50_, DC_100_, and DC_150_ respectively) (Fig. 4).

## Discussion

The objective of the study was to compare predictive and post-treatment dosimetry calculated at the voxel level based on ^99m^Tc-MAA SPECT and ^90^Y-microsphere PET respectively. Both global quantification and relative dose distribution deviations were analyzed.

In clinical routine, there is a bias between the ^90^Y activity planned to be delivered while performing predictive dosimetry and the activity delivered to the patient due to clinical hazards. This is mainly explained by the difficulty to predict the exact residual activity in the lines and the delay in time of injection. It can be noted that discrepancies in terms of activity values presented here could be easily translated into dose deviations.

^90^Y PET imaging feasibility and accuracy to assess microsphere distribution and perform post-treatment dosimetry has already been demonstrated [12, 16]. However, a deviation between the activity in the liver and the total activity in the PET’s FOV (− 20 ± 8%) was observed and seems to partly correspond to misplaced counts as described by Willowson et al. [17]. Moreover, the accuracy of reconstruction of the ^90^Y activity in PET images seems to be correlated to the patient’s BMI. The higher the BMI is, the smaller is the deviation between activity in the FOV and delivered activity but the higher is the activity in the FOV outside the segmented liver. These observations support the challenging ^90^Y PET quantification due to the very low ^90^Y internal pair production branching ratio (31.86 × 10^−6^) combined with the high random fraction. In these low true count statistics conditions, random and scatter corrections are more challenging resulting in noisy images and quantitative bias as reported by Carlier et al. [18]. These observations regarding both planned vs. delivered activity and ^90^Y PET quantification were not discussed by other authors in their dosimetry comparison papers where the total PET signal or the signal included in the body contours was rescaled to the administered activity [3, 5, 19].

Comparison between normalized ^99m^Tc-MAA SPECT and ^90^Y-microsphere PET, in terms of relative dose distribution, showed a good correlation and no significant difference was found. This result emphasizes the predictive value of ^99m^Tc-MAA SPECT-based dosimetry. However, perfect reproducibility of the radiological gesture is challenging. In our population, reproducibility of the catheter tip position between simulation and therapy was good in most of the procedures evaluated. However, dose distribution was significantly impacted when catheter tip position differed by a few millimeters between simulation and treatment (higher difference in terms of *D*_T_ and lower isodose Dice similarity).

Besides, for catheter position closer to a major artery bifurcation, *D*_T_ differences tended to be higher and isodose Dice similarity was lower. Dice coefficient calculated on the isodoses extracted from predictive and post-treatment dosimetry enabled to compare quantitatively spatial dose distribution. Reproducibility of catheter position and its distance to an artery bifurcation were shown as having an influence on dose distribution deviations. These results are in agreement with the literature [1, 20–22] and lead to two main recommendations. First, the catheter tip position should be reproduced as identical as possible and far from major bifurcation if possible. Second, on-table changes should not be made on the day of therapy without a new simulation stage.

Even if one could overcome these human factors, flow differences and differences inherent to the imaging modality used would likely induce unavoidable deviations between ^99m^Tc-MAA SPECT- and ^90^Y-microsphere PET-based dosimetry.

In addition to the radiological gesture previously discussed, other factors inherent to the particles differences and the injection procedure can influence particle biodistribution and consequently cause dose distribution variations. As mentioned by several authors [1, 4–6, 20, 21], the main ones are the number of particles injected, the injection volume and velocity, the particle physical properties (size/shape, density), the possible progress of disease, the occurrence of vasospasm during injection, etc. These factors cannot be easily quantified, hence analyzing their impact was beyond the scope of the present study.

Overall, both predictive and post-treatment dosimetry are necessary. The first one is essential to optimize activity planning by predicting dose to target and non-target volumes. The second one, in addition to visual PET vs. SPECT image comparison, is the only way to quantify potential discrepancies between the two procedures and assess actual absorbed doses (particularly in case of technical failure as defined by Kao et al. [6]).

Our findings support other studies, but all are limited by a small number of patients. A larger cohort is required to establish reliable confidence intervals of expectable mean dose quantitative metrics deviation between ^99m^Tc-MAA SPECT and ^90^Y-microsphere PET dosimetry. Moreover, it should be noted that this study is based exclusively on an HCC population treated with glass microspheres and the results may not be valid in different conditions.

To conclude, as mentioned by Garin et al., not only the use of MAA as a good surrogate of microsphere is controversial but also the whole SIRT simulation stage [23]. In recent years, ^166^Ho-microspheres (QuiremSpheres®, Quirem Medical B.V., Utrecht, The Netherlands) have been developed as an alternative to ^90^Y-microspheres. Their imaging properties, the ability to use a safe scout dose of the same particles for simulation as the ones used for therapy, and the possibility of a single day procedure lead us to expect promising results [24].

## Conclusions

Substantial deviations were observed between the activity measured on ^90^Y PET images and planned activity. This was likely related to clinical hazards and equipment issues, especially systematic bias in ^90^Y PET quantification due to low count and high random fraction. However, when comparing relative dose distributions between the tumor and the normal liver, a good correlation was observed between predictive and post-treatment dosimetry, highlighting the predictive value of ^99m^Tc-MAA SPECT dosimetry. Additionally, reproducibility of radiological gesture reduced variability, in agreement with the literature. To minimize dose distribution deviations, the catheter tip position should be reproduced as identically as possible and as far from major bifurcation as possible.

## Data Availability

Please contact the author for data requests.

## Abbreviations

BMI: Body mass index
FOV: Field of view
HCC: Hepatocellular carcinoma
MAA: Macroaggregated albumin
SIRT: Selective internal radiation therapy
